# Inhibition of CUG-binding protein 1 and activation of caspases are critically involved in piperazine derivative BK10007S induced apoptosis in hepatocellular carcinoma cells

**DOI:** 10.1371/journal.pone.0186490

**Published:** 2017-10-16

**Authors:** Ju-Ha Kim, Hee Young Kwon, Dong Hoon Ryu, Min-Ho Nam, Bum Sang Shim, Jin Han Kim, Jae Yeol Lee, Sung-Hoon Kim

**Affiliations:** 1 Cancer Molecular Targeted Herbal Research Center, College of Korean Medicine, Kyung Hee University, Seoul, Republic of Korea; 2 Research Institute for Basic Sciences and Department of Chemistry, College of Sciences, Kyung Hee University, Seoul, Republic of Korea; Institute of Biochemistry and Biotechnology, TAIWAN

## Abstract

Though piperazine derivative BK10007S was known to induce apoptosis in pancreatic cancer xenograft model as a T-type CaV3.1 a1G isoform calcium channel blocker, its underlying antitumor mechanism still remains unclear so far. Thus, in the present study, the antitumor mechanism of BK10007S was elucidated in hepatocellular carcinoma cells (HCCs). Herein, BK10007S showed significant cytotoxicity by 3-[4,5-2-yl]-2,5-diphenyltetra-zolium bromide (MTT) assay and anti-proliferative effects by colony formation assay in HepG2 and SK-Hep1 cells. Also, apoptotic bodies and terminal deoxynucleotidyl transferase (TdT) dUTP Nick End Labeling (TUNEL) positive cells were observed in BK10007S treated HepG2 and SK-Hep1 cells by 4',6-diamidino-2-phenylinodole (DAPI) staining and TUNEL assay, respectively. Consistently, BK10007S increased sub G_1_ population in HepG2 and SK-Hep1 cells by cell cycle analysis. Furthermore, Western blotting revealed that BK10007S activated the caspase cascades (caspase 8, 9 and 3), cleaved poly (ADP-ribose) polymerase (PARP), and downregulated the expression of cyclin D1, survivin and for CUG-binding protein 1 (CUGBP1 or CELF1) in HepG2 and SK-Hep1 cells. Conversely, overexpression of CUGBP1 reduced cleavages of PARP and caspase 3, cytotoxicity and subG_1_ population in BK10007S treated HepG2 cells. Overall, these findings provide scientific evidences that BK10007S induces apoptosis via inhibition of CUGBP1 and activation of caspases in hepatocellular carcinomas as a potent anticancer candidate.

## Introduction

Hepatocellular carcinoma (HCC) is one of intractable cancers worldwide and the fifth incidence in the United States according to The American Cancer Society [1, 2]. It is well documented that most of HCCs have been treated by hepatic resection, the chemotherapy and radiotherapy [3, 4]. Nevertheless, effective treatment of HCCs has limitations due to side effects, chemoresistance and recurrence. Thus, more effective therapy has been developed to enhance apoptotic efficacy and reduce chemoresistance. There are accumulating evidences that several survival genes such as MDR1, MRP, LRP [5, 6], survivin [7] and CUGBP1 [8] are critically involved in chemoresistance to chemotherapy. Among them CUGBP1, so called CELF1 (CUGBP Elav-like family member 1), is reported to be overexpressed in DM (Myotonic dystrophy) [9] and several cancer cells [10–14].

Piperazines (1,4 diazacyclohexane) are known nitrogen containing heterocyclic compounds that display a broad range of biological activities such as antiinflmmatory, antifungal, antimalarial and anticancer effects. Recently piperazine derivative BK10007S was known to show anticancer activity as a calcium channel blocker [15–17], since T-type calcium channel blockers are associated with proliferation of hepatocellular carcinoma [18, 19]. Nevertheless, its underlying antitumor mechanism has never been examined in hepatocellular carcinoma cells so far. Thus, in the current study, the apoptotic mechanism of BK10007S was elucidated in HepG2 and SK-Hep1 cells especially in association with CUGBP1.

## Materials and methods

### Cell culture

Cell lines were obtained from the American Type Culture Collection (ATCC; Manassas, VA, USA). HepG2 (ATCC^®^ HB-8065^™^) was cultured in low glucose Dulbecco’s modified Eagle medium (DMEM) supplemented with 10% fetal bovine serum (FBS; WelGENE, Daegu, South Korea) and 1% antibiotic-antimycotic solution containing 100 units/ml penicillin, 0.1 mg/ml streptomycin and 0.25 mg/ml amphotericin B (WelGENE, Daegu, South Korea), while SK-Hep1 (ATCC^®^ HTB52^™^), Hep3B (ATCC^®^ HB-8064^™^) and Panc-1 (ATCC^®^ CRL-1469^™^) was cultured in high glucose DMEM, and HCT116 (ATCC^®^ CCL-247^™^) and H460 (ATCC^®^ HTB-8064^™^) were cultured Roswell Park Memorial Institute (RPMI) 1640 with 10% FBS (WelGENE, Daegu, South Korea) and 1% antibiotic-antimycotic solution (WelGENE, Daegu, South Korea). Cell lines were maintained at 37°C with 5% CO2 in a humidified incubator and were used within 2 months of resuscitation.

### Chemicals and reagents

BK10007S was supplied to us by Jae Yeol Lee Ph.D. (Kyung Hee University, South Korea). Stock solution was prepared in dimethyl sulfoxide (DMSO; Ducksan, Ansan, South Korea). Anti-PARP (#9542, Cell Signaling Technology, Beverly, MA, USA), pro caspase 8 (#9746, Cell Signaling Technology, Beverly, MA, USA), procaspase 9 (#9502, Cell Signaling Technology, Beverly, MA, USA), cleaved caspase 3 (#9664, Cell Signaling Technology, Beverly, MA, USA) and cyclin D1 (#2978, Cell Signaling Technology, Beverly, MA, USA) were purchased from Cell Signaling Technology. Anti-survivin (#SC-17779, Santa Cruz, Dallas, TX, USA) and CUGBP1 (#SC-20003, Santa Cruz, Dallas, TX, USA) antibodies were obtained from Santa Cruz. Anti-β-actin (#A2228, St Louis, MO, USA) antibody was purchased from Sigma-Aldrich (Sigma-Aldrich, St Louis, MO, USA).

### Cytotoxicity assay

MTT (3-(4,5-dimethylthiazol-2-yl)-2,5-diphenyltetrazolium bromide) assay was performed to evaluate the cytotoxicity of the BK10007S in HepG2 and SK-Hep1 cells. HCCs were seeded in 96-well culture plates at a density of 1×10^4^ cells/well and incubated overnight. The cells were exposed to various concentrations (0, 3, 7 and 8.5μM) of BK10007S for 24 h. 50 μl of MTT (1mg/ml, Sigma-Aldrich, St Louis, MO, USA) was added to each well for 1 h at 37°C in dark and then the optical density was measured with Microplate Reader (TECAN, Mannedorf, Switzerland) at 570 nm.

### Colony formation assay

HepG2 and SK-Hep1 cells (1×10^3^ cells/well) were seeded onto 6-well cell culture plate to examine long term anti-proliferative effect of BK10007S. Cells were treated with BK10007S (0, 7 and 8.5 μM) for 24 h and the media was changed every three days. Cells were incubated for 2 weeks to grow colonies at 37°C under the condition of 5% CO_2_, fixed with methanol and stained with crystal violet solution (w/v 25% crystal violet, w/w 75% methanol) and dried overnight. The wells were detected by EOS 450D Canon digital camera (Canon, Tokyo, Japan). Optical density (OD) was measured by Microplate Reader (TECAN, Mannedorf, Switzerland) at 590 nm.

### Cell cycle analysis

For evaluation of apoptotic sub G_1_ portion, HepG2 and SK-Hep1 cells (4×10^5^ cells/well) were seeded onto 6-well cell culture plate and were exposed to BK10007S for 24 h. The cells were harvested by trypsinization, washed with cold PBS, and then fixed with 75% ethanol at 20°C overnight. The completely fixed cells was collected, washed with PBS and incubated with RNase (1 mg/ml) for 1 h at 37°C. Then the cells were stained with 500 μl Propidium Iodide (PI; 25 μg/ml, Sigma-Aldrich, St Louis, MO, USA) at room temperature. The stained cells were analyzed by FACS Calibur flow cytometer (Becton Dickinson, FranklinLakes, NJ, USA).

### DAPI staining assay

HepG2 and SK-Hep1 cells were treated with BK10007S to identify the fragmentation and condensation of the nucleus of HepG2 and SK-Hep1 cells. The cells were fixed with 4% paraformaldehyde, stained with 25 μg/ml DAPI. Stained cells were mounted with mounting medium (Vectashield, CA, USA) and visualized by using Olympus FLUOVIEW FV10i confocal microscope (Olympus, Tokyo, Japan).

### TUNEL assay

HepG2 cells in a four well chamber were treated with BK10007S for 24 h to confirm the apoptotic bodies in BK10007S treated HepG2 cells. The cells were fixed with 4% paraformaldehyde and permeabilized in PBS with 0.1% Triton X-100 and 0.1% sodium citrate for 2 min on ice. 50 μl TUNEL reaction mixture (Roche, Mannheim, Germany) was added to the sample slide, which was covered with cover-film and incubated in a humidified atmosphere for 1 h at 37°C. Then the stained cells were rinsed twice with PBS, stained with 25 μg/ml PI (Sigma-Aldrich, St Louis, MO, USA), mounted with the mounting medium (Vectashield, CA, USA) and visualized by using Olympus FLUOVIEW FV10i confocal microscope (Olympus, Tokyo, Japan).

### Western blotting

HepG2 and SK-Hep1 cells were lysed in RIPA buffer (Sigma-Aldrich, St Louis, MO, USA) containing 50 mM Tris- HCl, 150 mM NaCl, 2 mM EDTA, and 1% TritonX-100 with protease inhibitors (Roche, Mannheim, Germany) and phosphatase inhibitors (Sigma-Aldrich, St Louis, MO, USA). The lysates were quantified by DC Protein Assay Kit II (Bio-Rad Hercules, CA, USA). The protein samples were electrophoresed on 8 to 15% SDS-polyacrylamide gels, and transferred to nitrocellulose membranes. Membranes were blocked with TBST diluted 5% skim milk for 1h at room temperature or TBST diluted 5% BSA for 4 h at 4°C. Then these were incubated with primary antibodies diluted in 5% BSA in TBST overnight at 4°C, washed three times with TBST, and incubated with HRP-conjugated secondary antibodies for 1 h. Expression was visualized by using ECL Immunoblotting detection reagent (GE Healthcare, Buckinghamshire, UK).

### Transfection assay

Flag-tagged CUGBP1 was obtained from prof. JH Ha. Ph.D (Kyung Hee University, South Korea). For DNA transient-overexpression transfection, HepG2 cells were transfected with flag-tagged CUGBP1 plasmid using transfection reagent (iN-fect, iNtRON Biotechnology, Seongnam, South Korea) for further experiment.

### Statistical analysis

All statistical analyses were carried out by using SPSS (SPSS, IBM Corporation) and GraphPad Prism software (Version 5.0, California, USA). Data were expressed as means ± SD from at least three independent experiments. The one-way analysis of variance (ANOVA) followed by a Turkey post-hoc test was used for within-group comparisons. For two group comparisons, the independent sample Student’s *t*-test was performed. A significant difference was considered if the *p*-value was less than 0.05.

## Results

### BK10007S exerts cytotoxic and antiproliferative effects in HepG2 and SK-Hep1 HCCs

**Fig 1:**
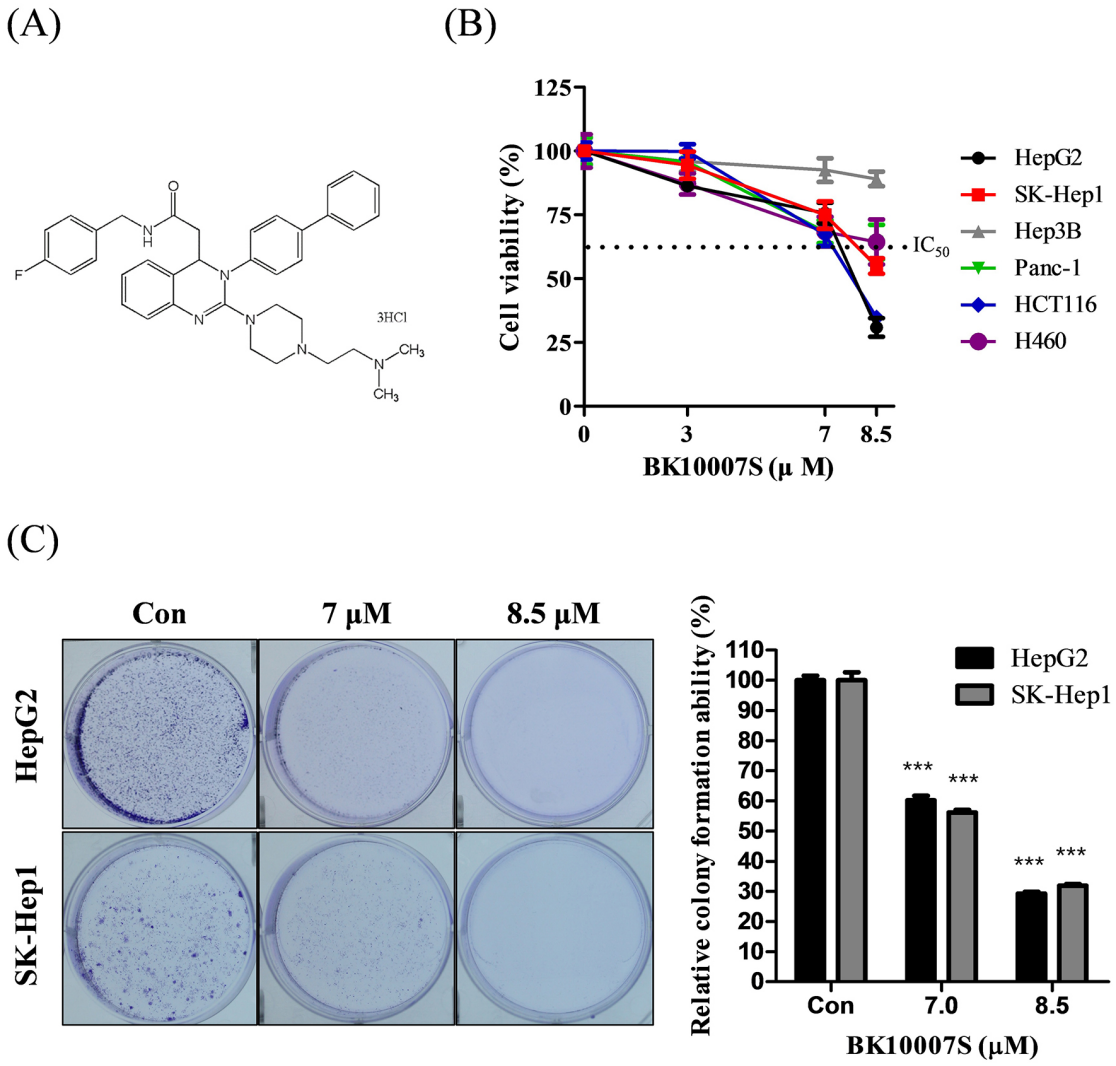
The chemical structure and cytotoxic and antiproliferative effects of BK10007S in HepG2 and SK-Hep1 cells. (A) The chemical structure of BK10007S (C_37_H_44_Cl_3_FN_6_O). (B) Cytotoxicity of BK10007S in HepG2, SK-Hep1, Hep3B, Panc-1, HCT116 and H460 various cancer cells. The cells were treated with various concentrations of BK10007S (0, 3, 7 and 8.5 μM). Cell viability was measured by MTT assay. Data represent means ± S.D from three independent experiments. (C) Anti-proliferative effect of BK10007S in HepG2 and SK-Hep1 cells by colony formation assay. HepG2 and SK-Hep1 cells (1 x 10^3^ cells) were treated with various concentrations (0, 7 and 8.5 μM) of BK10007S for 24 h and culture media were changed every three days. Cells were incubated for 2 weeks to grow colonies at 37°C under the condition of 5% CO2, fixed with methanol, stained with crystal violet solution and dried overnight. The wells were detected by EOS 450D Canon digital camera. *** *p* < 0.001 vs control. Data represent means ± S.D from three independent experiments.

MTT assay was performed to test the cytotoxic effect of BK10007S (Fig 1A) in HepG2, SK-Hep1, Hep3B (Hepatocellular carcinoma cell lines), Panc-1 (Pancreatic cancer cell line), HCT116 (Colorectal cancer cell line) and H460 (Lung cancer cell line) various cancer cell lines. As shown in Fig 1B, BK10007S significantly decreased the viability of HepG2 and SK-Hep1 cells in a dose dependent manner. Also, BK10007S attenuated the proliferative effect by reducing the size and number of colonies compared to untreated control in HCCs by colony formation assay (Fig 1C).

### BK10007S induces apoptotic morphological changes in HepG2 and SK-Hep1 HCCs

**Fig 2:**
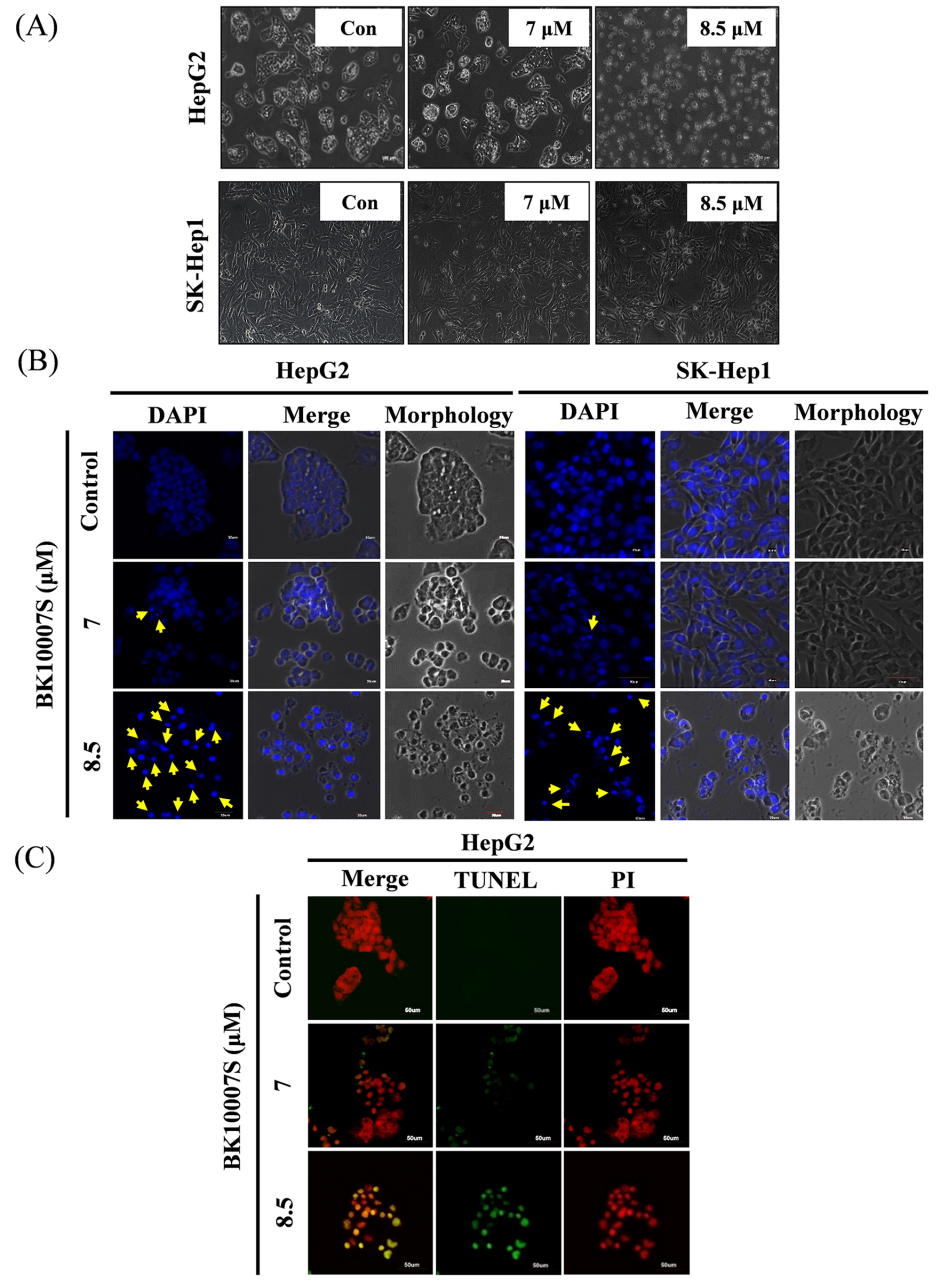
Apoptotic morphological changes of BK10007S in HepG2 and SK-Hep1 cells. (A) Detached and dead cells were observed in BK10007S-treated HepG2 and SK-Hep1 cells by optical microscope (Zeiss Observer A1). (B) HepG2 and SK-Hep1 cells were treated with 7 and 8.5 μM concentrations of BK10007S for 24 h, washed with PBS and stained with 50 μg/ml of DAPI. Apoptotic bodies stained by DAPI were observed. (C) HepG2 cells were treated with BK10007S for 24 h and stained with PI and TUNEL according to Roche’s protocol. This observation was detected by using Olympus FLOVIEW FV10i confocal microscope.

BK10007S induced apoptotic morphological changes by reducing cell to cell contact (Fig 2A), showing apoptotic bodies and fragmentation of nucleus in HepG2 and SK-Hep1 cells by DAPI staining (Fig 2B) and also increased the number of TUNEL positive green fluorescent cells in HepG2 cells (Fig 2C).

### BK10007S increases sub G_1_ population and regulates apoptosis-related proteins and CUGBP1 in HepG2 and SK-Hep1 HCCs

**Fig 3:**
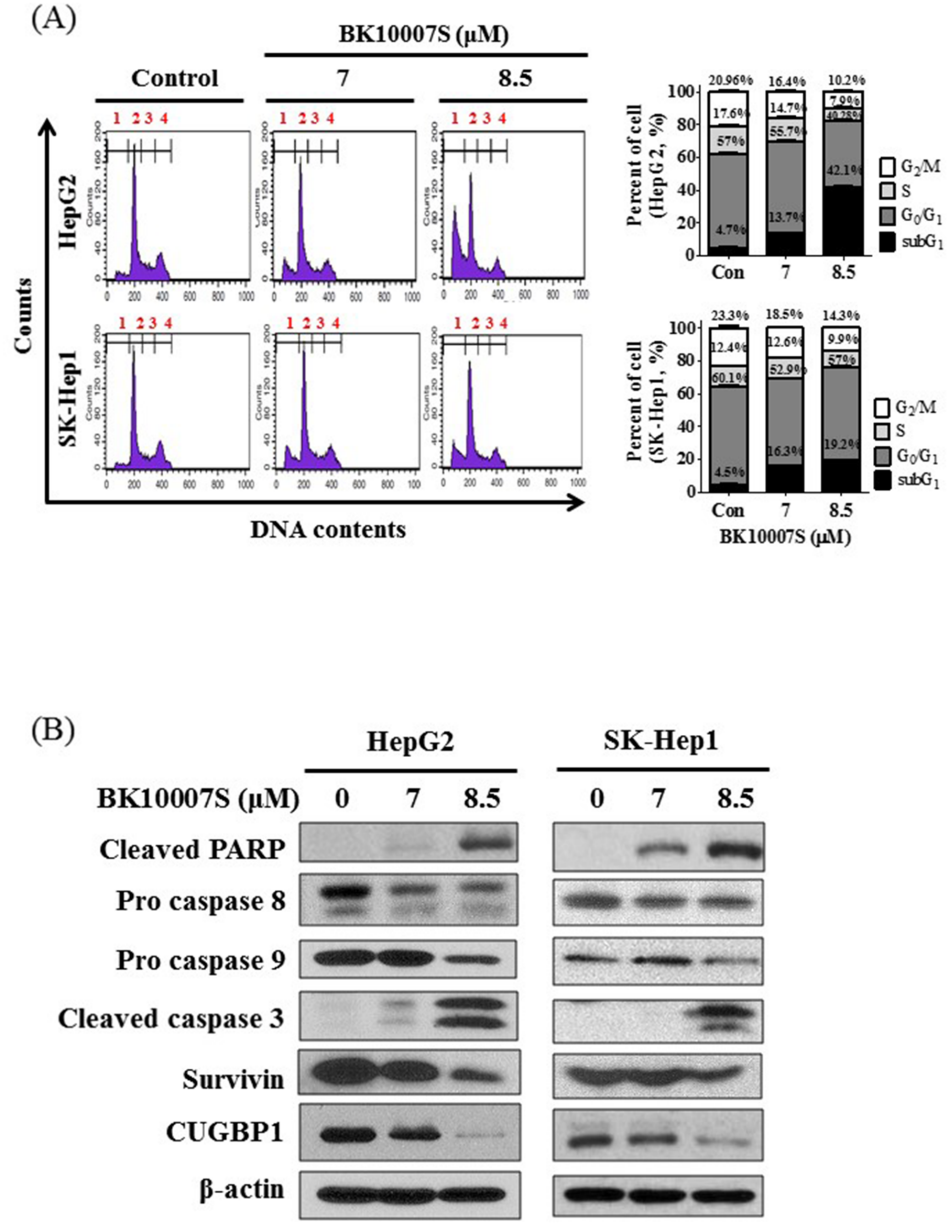
Effect of BK10007S on sub G1 population and apoptosis related proteins in HepG2 and SK-Hep1 cells. (A) HepG2 and SK-Hep1 cells were treated with 7 or 8.5 μM of BK10007S for 24 h and fixed with 70% ethanol overnight. The cells were stained with 50 μg/ml of PI and then analyzed by FACS Calibur using CellQuest software. Data represent means ± S.D from three independent experiments (*** *p* < 0.001 vs control). (B) HepG2 and SK-Hep1 cells were treated with 7 and 8.5 μM of BK10007S for 24 h and were subjected to Western blotting for PARP, procaspase-8, procaspase-9, cleaved caspase-3, survivin and CUGBP1. Membranes were probed with a β-actin antibody as a loading control.

To confirm apoptotic portion at cellular level, cell cycle analysis was performed in HepG2 and SK-Hep1 cells after treatment of BK10007S (0, 7, and 8.5 μM) for 24 h. Here BK10007S effectively increased sub G_1_ population in HepG2 and SK-Hep1 cells, but HepG2 cells were more susceptible to BK10007S compared to SK-Hep1 cells at 8.5 μM (Fig 3A). Next, after exposure to BK10007S for 24 h, Western blotting was conducted for cleaved PARP, pro caspase 8, pro caspase 9, cleaved caspase 3, survivin and CUGBP1. As shown in Fig 3B, BK10007S cleaved PARP and caspase 3, attenuated the expression of procaspase 8/9, survivin and CUGBP1 in two HCCs. Also, phosphorylation of AKT and ERK and the expression of Cyclin D1 were attenuated in SK-Hep1 cells by BK10007S, while it did not affect phosphorylation of JNK and p38 MAPK (S1 Fig).

### Overexpression of CUGBP1 disturbs BK10007S induced apoptosis in HCCs

**Fig 4:**
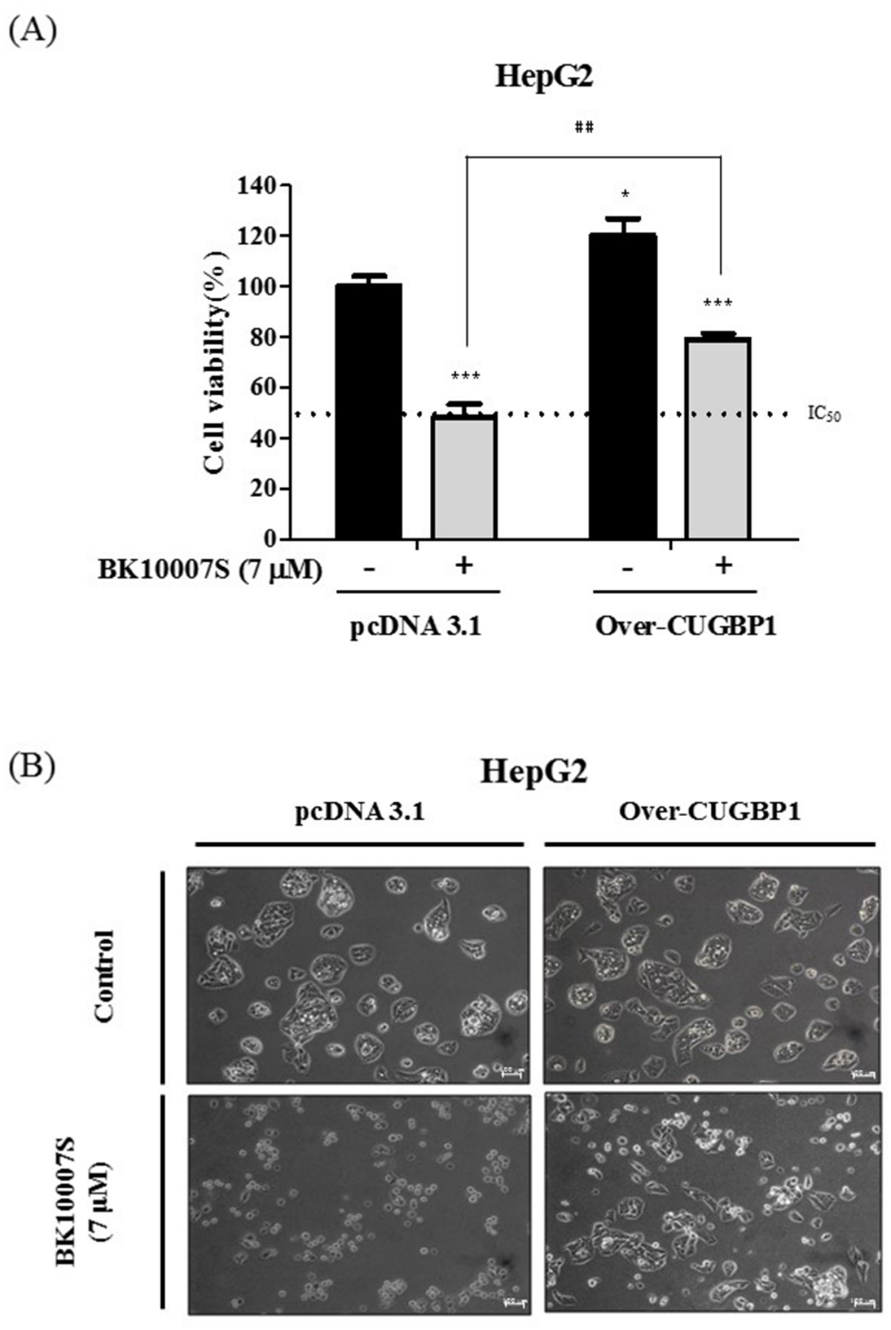
Effect of CUGBP1 overexpression on cell viability and apoptotic morphology in BK10007S treated HepG2 cells. (A) HepG2 cells were transfected with pcDNA 3.1 plasmid or CUGBP1-overexpression plasmid for 24 h and exposed to BK10007S (0 and 7 μM) for 24 h. Cell viability was measured by MTT assay. Data represent means ± S.D from three independent experiments. * *p* < 0.05, ^##^ *p* < 0.001 vs pcDNA 3.1 control. (B) Morphological changes were observed by Zeiss observer A1 (Zeiss, Oberkochen, Germany) in HepG2 cells transfected with pcDNA 3.1 plasmid or CUGBP1 overexpression plasmid following exposure to BK10007S (0 and 7 μM) for 24 h.

To identify whether overexpression of CUGBP1 blocks apoptosis by BK10007S, cytotoxicity assay was conducted in HepG2 cells exposed to 7 μM of BK10007S for 24 h. overexpression of CUGBP1 repressed the cytotoxic effect by BK10007S in HepG2 cells as shown in Fig 4A. It was also observed that cell death and anoikis by BK10007S were significantly overcome in CUGBP1-overexpressed HepG2 cells (Fig 4B). Also, overexpression of CUGBP1 enhanced the proliferation of HepG2 cells and blocked antiproliferative activity of BK10007S in HepG2 cells (S2 Fig).

**Fig 5:**
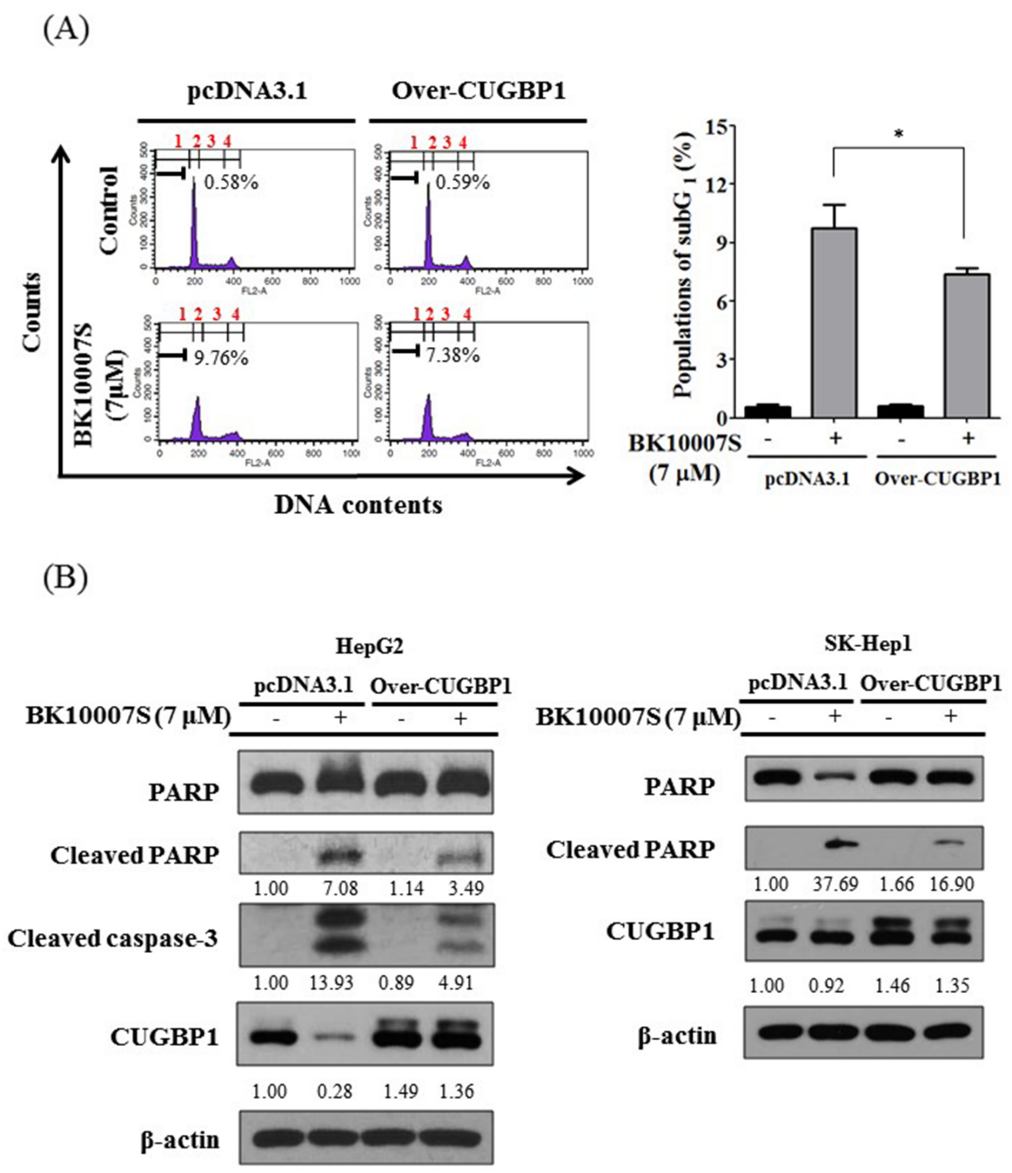
Effect of CUGBP1 overexpression on subG_1_ population, cleaved PARP and cleaved caspase-3 in BK10007S treated HCCs. (A) HepG2 cells transfected with pcDNA 3.1 plasmid or CUGBP1 overexpression plasmid were exposed to 7 μM of BK10007S for 24 h and cell cycle analysis was conducted by FACS Calibur using PI staining. Data represent means ± S.D from three independent experiments (* *p* < 0.05 vs pcDNA 3.1 control). (B) HepG2 and SK-Hep1 cells transfected with pcDNA 3.1 plasmid or CUGBP1 overexpression plasmid were exposed to 7 μM of BK10007S for 24 h and were subjected to Western blotting for PARP, cleaved PARP, cleaved caspase 3 and CUGBP1. Membranes were probed with a β-actin antibody as a loading control.

Consistently, overexpression of CUGBP1 reduced sub G_1_ accumulation by BK10007S in HepG2 cells compared to untreated control as shown in Fig 5A. Furthermore, protein expression of cleaved PARP and cleaved caspase 3 was decreased in CUGBP1 overexpressed-HepG2 and SK-Hep1 cells compared to pcDNA 3.1 vector control (Fig 5B).

## Discussion

To develop more potent anticancer agents for HCC treatment, the underlying apoptotic mechanism of piperazine derivative BK10007S was elucidated in HepG2 and SK-Hep1 cells. Herein BK10007S showed significant cytotoxicity and reduced the size and number of colonies in HepG2 and SK-Hep1 cells by MTT and clonogenic assays, implying significant antitumor effect of BK10007S via cytotoxic and anti-proliferative effects of BK10007S.

Apoptosis consists of typical morphological and biochemical changes. The morphological events are initiated by cell shrinkage and chromatin condensation leading to membrane blebbing, nuclear fragmentation and apoptotic body formation [20]. Herein BK10007S reduced cell to cell contact and also DAPI staining and TUNEL assay revealed that more apoptotic bodies and TUNEL positive cells were shown in BK10007S treated HepG2 and/or SK-Hep1 cells, indicating apoptotic property of BK10007S in HCCs. Consistently, BK10007S increased subG_1_ apoptotic portion in HepG2 and SK-Hep1 cells by flow cytometric analysis.

Two typical apoptotic pathways are the intrinsic mitochondrial-dependent pathway and the extrinsic death receptor-mediated pathway via caspase activation [21, 22]. In the intrinsic pathway, mitochondrial cytochrome C is released into the cytosol to form apoptosome with apoptotic protease activating factor 1(Apaf-1) to activate caspase 9 [23]. In contrast, in the extrinsic pathway, stimulation of death receptors such as TNF-related apoptosis-inducing ligand (TRAIL) receptors or CD95 (APO-1/Fas) results in the activation of the initiator caspase 8, leading to activation of effector caspase 3 for apoptosis induction [24]. Here BK10007S activated the expression of caspase 8, 9 and 3, and cleavage of PARP, and also suppressed anti-apoptotic protein survivin in HepG2 and SK-Hep1 cells, demonstrating intrinsic and extrinsic apoptotic pathway of BK10007S in HCCs.

Emerging evidences reveal that CUGBP1 is involved in cell proliferation, growth and cell cycle [14], mostly overexpressed in glioma [25] and oral squamous cell carcinoma [26] and acts as a target molecule for brain metastasis from non-small lung cancer cells [27]. Also, previous evidences showed that the ectopic expression of CUGBP1 increases resistance to caspase dependent apoptosis in various cells such as nhESO esophageal epithelial cells [28] and oral cancer cells [10]. Similarly, current study showed overexpression of CUGBP1 suppressed the cytotoxicity, sub G_1_ accumulation and cleavages of PARP and caspase 3 induced by BK10007S in HepG2 cells, indicating the pivotal role of CUGBP1 in BK10007S induced apoptosis in HCCs.

However, it was well documented that AKT induces CUGBP1, leading to increase its affinity to cyclin D1 at mRNA [29, 30] and protein levels [31]. Also, CUGBP1 is closely related to p-ERK in colorectal cancer cells [32]. Here BK10007S reduced the expression of p-AKT, Cyclin D1 and p-ERK, but not p38MAPK/p-JNK in SK-Hep1 cells, implying the involvement of PI3K/AKT, ERK or cyclin D1 in BK10007S induced apoptosis in HCCs regardless of CUGBP1 signaling. Hence, the detailed mechanism of CUGBP1 along with upstream and downstream molecules via protein-protein interactions should be further studied in the future.

## Conclusions

Our results demonstrate that a calcium channel blocker BK10007S exerted cytotoxic and anti-proliferative effects, increased sub G_1_ population and induced apoptosis via inhibition of CUGBP1 and activation of caspases in HepG2 and SK-Hep1 hepatocellular carcinomas. Overall, these findings suggest that BK10007S induces apoptosis via inhibition of CUGBP1and activation of caspases in HCCs as a potent anticancer agent.

## Supporting information

S1 Fig Effect of BK10007S on phosphorylation of AKT and MAPKs and the expression of Cyclin D1 in SK-Hep1 cells. (TIF)

S2 Fig Overexpression of CUGBP1 reduces the antiproliferative activity of BK10007S in HepG2 cells by colony formation assay. (TIF)
