# Supplementary figures and images for: Inhibition of CUG-binding protein 1 and activation of caspases are critically involved in piperazine derivative BK10007S induced apoptosis in hepatocellular carcinoma cells
Source: PLoS One. 2017 Oct 16;12(10):e0186490. doi: 10.1371/journal.pone.0186490 (PMC5643113; doi:10.1371/journal.pone.0186490)

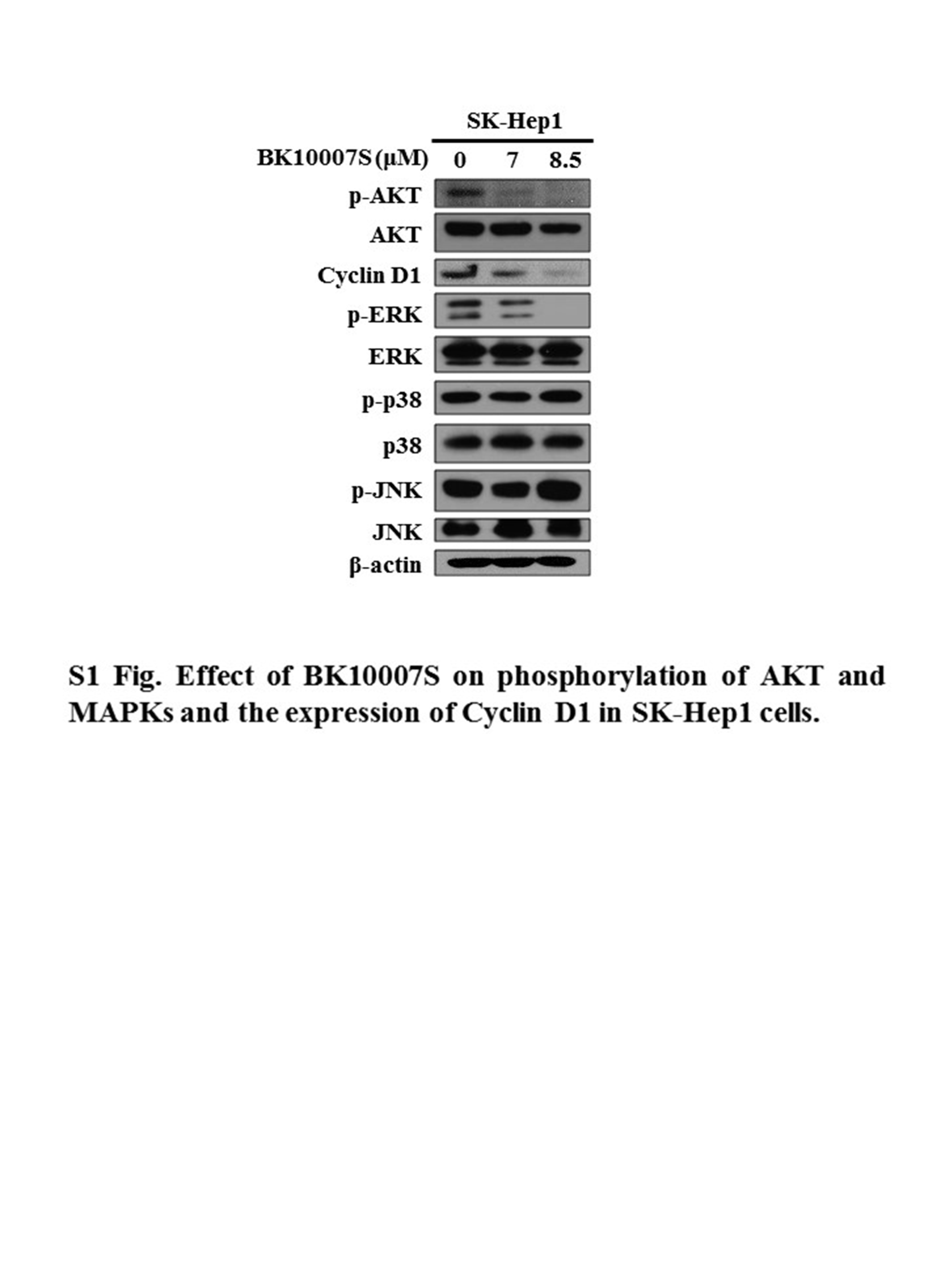

Supplement: S1 Fig — (TIF) [file pone.0186490.s001.tif]

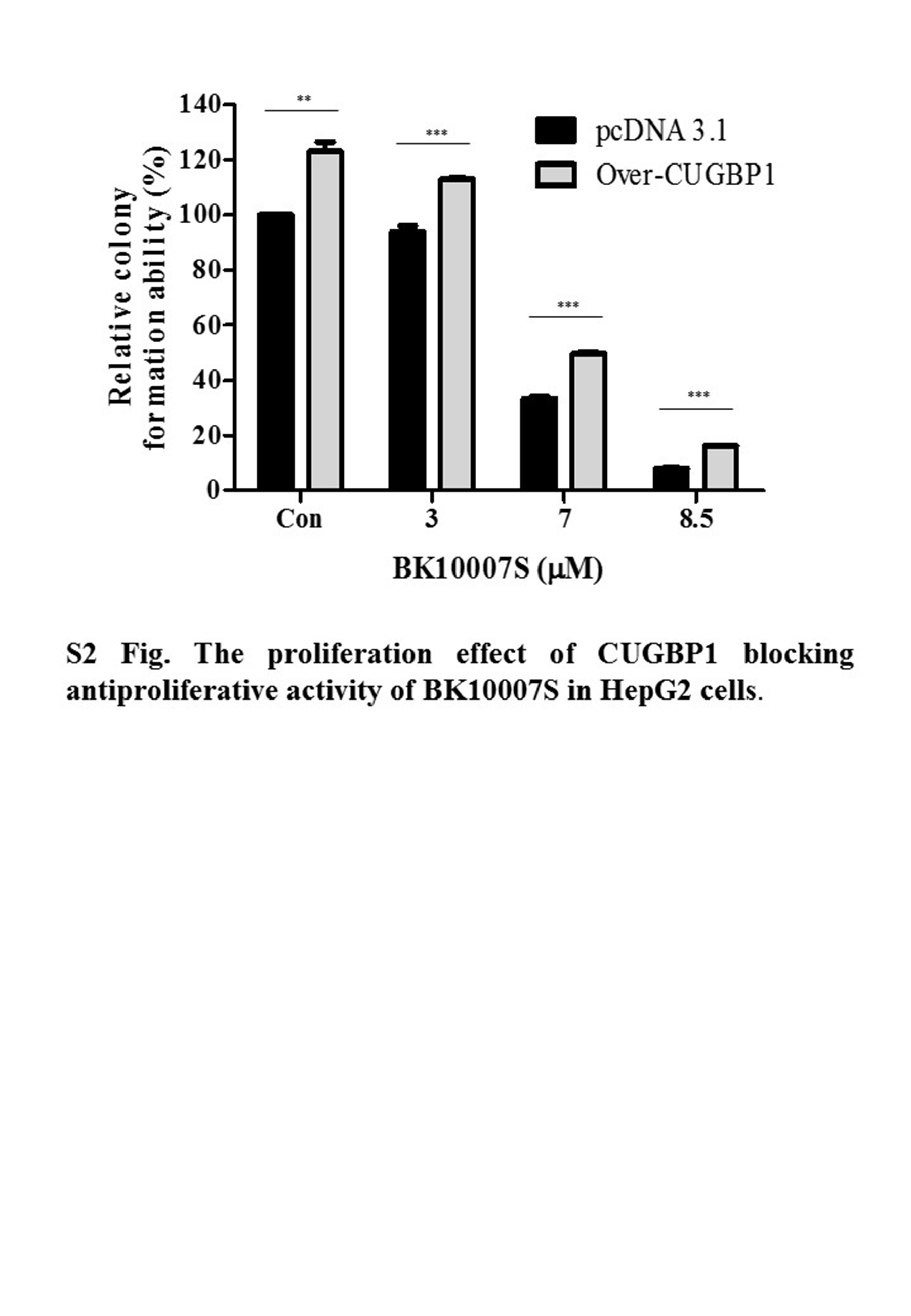

Supplement: S2 Fig — (TIF) [file pone.0186490.s002.tif]
